# *Prph2* knock-in mice recapitulate human central areolar choroidal dystrophy retinal degeneration and exhibit aberrant synaptic remodeling and microglial activation

**DOI:** 10.1038/s41419-023-06243-8

**Published:** 2023-11-01

**Authors:** María José Ruiz-Pastor, Xavier Sánchez-Sáez, Oksana Kutsyr, Henar Albertos-Arranz, Carla Sánchez-Castillo, Isabel Ortuño-Lizarán, Natalia Martínez-Gil, Lorena Vidal-Gil, Lucía Méndez, Manuel Sánchez-Martín, Victoria Maneu, Pedro Lax, Nicolás Cuenca

**Affiliations:** 1 Physiology, Genetics, and Microbiology, University of Alicante, Alicante, Spain; 2 Optics, Pharmacology, and Anatomy, University of Alicante, Alicante, Spain; 3 Transgenic Facility and Department of Medicine, University of Salamanca, Salamanca, Spain; 4 Institute for Biomedical Research of Salamanca (IBSAL), Salamanca, Spain; 5 Alicante Institute for Health and Biomedical Research (ISABIAL), Alicante, Spain

**Keywords:** Neurodegeneration, Retina

## Abstract

Central areolar choroidal dystrophy is an inherited disorder characterized by progressive choriocapillaris atrophy and retinal degeneration and is usually associated with mutations in the *PRPH2* gene. We aimed to generate and characterize a mouse model with the p.Arg195Leu mutation previously described in patients. Heterozygous (*Prph2*^*WT/KI*^) and homozygous (*Prph2*^*KI/KI*^) mice were generated using the CRISPR/Cas9 system to introduce the p.Arg195Leu mutation. Retinal function was assessed by electroretinography and optomotor tests at 1, 3, 6, 9, 12, and 20 months of age. The structural integrity of the retinas was evaluated at the same ages using optical coherence tomography. Immunofluorescence and transmission electron microscopy images of the retina were also analyzed. Genetic sequencing confirmed that both *Prph2*^*WT/KI*^ and *Prph2*^*KI/KI*^ mice presented the p.Arg195Leu mutation. A progressive loss of retinal function was found in both mutant groups, with significantly reduced visual acuity from 3 months of age in *Prph2*^*KI/KI*^ mice and from 6 months of age in *Prph2*^*WT/KI*^ mice. Decreased amplitudes in the electroretinography responses were observed from 1 month of age in *Prph2*^*KI/KI*^ mice and from 6 months of age in *Prph2*^*WT/KI*^ mice. Morphological analysis of the retinas correlated with functional findings, showing a progressive decrease in retinal thickness of mutant mice, with earlier and more severe changes in the homozygous mutant mice. We corroborated the alteration of the outer segment structure, and we found changes in the synaptic connectivity in the outer plexiform layer as well as gliosis and signs of microglial activation. The new *Prph2*^*WT/KI*^ and *Prph2*^*KI/KI*^ murine models show a pattern of retinal degeneration similar to that described in human patients with central areolar choroidal dystrophy and appear to be good models to study the mechanisms involved in the onset and progression of the disease, as well as to test the efficacy of new therapeutic strategies.

## Introduction

Monogenic hereditary retinal dystrophies, in which only one gene carries a mutation, are an attractive target for developing new gene therapies and neuroprotection strategies, which have the potential to be further extrapolated to other retinal dystrophies. Central areolar choroidal dystrophy (CACD) is a monogenic hereditary disease that involves progressive retinal degeneration [1]. CACD is considered a rare disease, and its prevalence is 1–9 cases per 100,000 people [2]. Patients with CACD show a gradual loss of visual acuity (VA) and color perception between the ages of 30 and 60 [3, 4]. CACD is caused by different mutations in the peripherin-2 gene (*PRPH2*), usually with an autosomal dominant inheritance pattern [5]. *PRPH2* encodes the glycoprotein peripherin-2 (PRPH2), also known as retinal degeneration slow (RDS) protein, or tetraspanin-22 [6]. PRPH2 is essential for the morphogenesis and structure of the outer segment (OS) discs of photoreceptor cells [7]. CACD presents high genetic heterogeneity [8], and at least five different mutations in *PRPH2* have been histologically and functionally described as causing this disease [3, 4, 9–13]. In Spain, there is a large family with a *PRPH2* gene mutation, p.Arg195Leu, which has been transmitted over at least 11 generations, with the first affected member dated in 1756. In this family, 119 relatives were reported to have retinal problems, and 56 living patients were diagnosed with CACD, 10 of whom were further confirmed by genetic testing. The CACD phenotype of four members of this family was previously characterized by our group [14]. To assess the efficiency of different therapeutic approaches, a deep knowledge of the pathophysiology of CACD and the progression of cellular and molecular changes in the retina is essential. Thus, we addressed, using the CRISPR/Cas9 system, the generation of a mouse model carrying the same point mutation as this Spanish family. With this mouse model, we characterized the progression of retinal degeneration that affects this family with CACD.

## Material and methods

### Generation of the *P*RPH*2* p.Arg195Leu murine model using CRISPR/Cas9

#### CRISPR/Cas9 system design

The murine model C57BL/6J-*Prph2*^em1Sal^ carrying the mutation p.Arg195Leu was generated using CRISPR/Cas technology. One sgRNA (*Prph2*-sgRNA1) overlapping the intron 1-exon 2 genomic sequence of *Prph2* was designed (http://bioinfogp.cnb.csic.es/tools/breakingcas/) to induce a double-strand break at the c.584 position. Two complementary oligos corresponding to *Prph2*-sgRNA1 were designed, including two 4 bp overhang sequences, to clone both into the pX458 vector (Addgene plasmid # 48138). The pX458 vector was linearized with BpiI (New England Biolabs Inc., Ipswich, MA, USA), and the *Prph2*-sgRNA1 complementary oligos were denatured at 95 °C for 5 min, ramp-cooled to 25 °C over 45 min to allow annealing, and ligated with the linearized pX458. Competent cells were transformed with 2 μl of the ligated plasmid, and single colonies were grown before plasmid extraction (Qiagen, Hilden, Germany). A 200 nt single-stranded DNA oligonucleotide (ssODN) (Integrated DNA Technologies Inc., Coralville, IA, USA) containing the mutated sequence c.584 G > T (CGC > CTC, Arg>Leu) was designed as a template for homologous recombination to integrate the point alteration. The sgRNA sequence was PCR-amplified from the pX458-based vector with primers carrying the T7 RNA polymerase promoter at the 5′ ends. PCR products were purified (NZYGelpure kit, NZYTech, Lisboa, Portugal) and used as a template for in vitro T7 RNA polymerase transcription (MEGAshortscript T7 Transcription Kit, Thermo Fisher, Waltham, MA, USA). Ribonucleocomplexes were formed by incubating 200 ng of sgRNAs and 200 ng of Cas9 nuclease (Integrated DNA Technologies Inc.) at 37 °C for 10 min.

#### Mouse embryo microinjection

Then, 20 ng/µl sgRNA-Cas9 protein and 25 ng/µl ssODN template solution were microinjected into the pronucleus of one-cell stage embryos. These embryos were obtained from superovulated C57BL/6J females. Microinjected embryos were grown overnight on KSOM with nonessential amino acids (Millipore, Burlington, MA, USA) at 37 °C in a 5% CO_2_ atmosphere. After 12 h of culture, two-cell-stage embryos were recovered and transferred to foster CD1 females.

#### Analysis of CRISPR/Cas9 target sites

Founder mice were crossed with wild-type C57BL/6J animals to eliminate possible off-target effects. The inheritance of the alleles followed Mendelian principles. Pure knock-in heterozygotes (*Prph2*^*WT/KI*^) of the F3 generation were then crossed to obtain knock-in homozygous (*Prph2*^*KI/KI*^) mice. To check that these animals carried the point mutation p.Arg195Leu, we genotyped the animals using PCR and Sanger sequencing from DNA obtained from a biopsy of the tail. Animals for this study were obtained by cross-breeding.

#### Animals

All animals, wild-type (*Prph2*^*WT/WT*^), *Prph2*^*WT/KI*^, and *Prph2*^*KI/KI*^, were bred in the animal facility of the University of Alicante and maintained under the same environmental conditions: temperature of 23 ± 1 °C, humidity of 55–60%, photoperiod of 12/12 h light/dark, and light intensity of 300 lux. The retinal function of animals from the three genotypes was evaluated at 1, 3, 6, 9, 12, and 20 months of age using the optomotor test and electroretinography (ERG). Optical coherence tomography (OCT) was also performed at the same ages to evaluate the retinal structure. The number of animals used was *N* = 12 for optomotor testing, *N* = 11 for ERG, and *N* = 9 for OCT in each experimental group. We always ensured a randomized distribution of the animals in each age group and an equal number of females and males for each experiment. All procedures were approved by the Ethical Committee of the University of Alicante (2019/VSC/PEA/0266) and performed following EU Directive 2010/63/EU. For all procedures where anesthetization was required, animals were anesthetized with 100 mg/kg ketamine and 8 mg/kg xylazine. Once the procedures were finished, the mice were euthanized by cervical dislocation.

#### Optomotor test

To evaluate the VA of the animals, the Argos optomotor system was used (Instead Technologies, Elche, Spain). Mice were stimulated with a vertically oriented grating that rotated horizontally either in one sense or in the opposite, aleatory, for 5 s. The initial spatial frequency was 0.088 cycles/degree, and the contrast was 100%. Smooth head movements in the direction of the rotating grating were evaluated by a trained observer. The highest spatial frequency to which the mouse responded was considered the VA threshold of the animal.

#### Electroretinography

ERG responses were recorded in total darkness after overnight adaptation using the HMsERG LAB System (OcuScience, Henderson, NV, USA). The animals were anesthetized and kept at 38 °C on a thermal blanket. For pupil dilatation, 1% tropicamide (Alcon Cusí, Barcelona, Spain) was used. Additionally, a 0.2% polyacrylic acid carbomer gel (Novartis, Barcelona, Spain) was used to prevent eye dehydration and ensure the fitting of the recording electrodes. Fourteen stimuli with increasing luminance (−5.5 to 1 log cd s/m^2^) were presented to the animals. After a period of light adaptation, photopic responses were obtained using 8 increasing stimuli (−2.5 to 0.9 log cd s/m^2^). The amplitude of the a-wave was measured from the baseline to the most negative trough, and the amplitude of the b-wave was measured from the trough of the a-wave to the peak of the b-wave.

#### Optical coherence tomography

The retinal structure of the animal models was evaluated in vivo using the Spectralis OCT system (version 6.9.4.0, Heidelberg Engineering, Heidelberg, Germany). After anesthesia induction, pupils were dilated with 1% tropicamide (Alcon Cusí), and dehydration of the cornea was prevented by applying a drop of physiological serum in each eye. A high-resolution dense scan at the superior central region of the optic nerve was obtained at 1, 3, 6, 9, 12, and 20 months. The thickness of the total retina, including the retinal pigment epithelium (RPE), the outer nuclear layer (ONL), and the inner nuclear layer (INL), was measured at 400 μm from the optic nerve at ten different points separated by 200 μm using ImageJ [15]. Two different researchers performed the analysis in a double-blinded manner.

#### Immunofluorescence

After sacrificing the animals, the dorsal margin of the limbus was marked by a suture (Lorca Marín, Murcia, Spain). The eyes were enucleated and fixed in 4% paraformaldehyde for 1 h, washed in 0.1 M phosphate buffer (PB), pH 7.4, and cryoprotected by sucrose gradient (15, 20, and 30%). The cornea, lens, and vitreous humor were removed, and the eyecups were embedded in Tissue-Tek OCT compound (Sakura Finetech, Tokyo, Japan) and frozen in liquid nitrogen. Serial sections 15 µm thick were obtained using a cryostat (CM1950; Leica Microsystems, Wetzlar, Germany), mounted on microscope slides (Menzel GmbH & Co KG, Braunschweig, Germany), air-dried, and stored at −20 °C.

For the immunostaining procedure, sections were thawed, washed in PB, and incubated at room temperature for 1 h in blocking solution consisting of 10% (v/v) normal donkey serum in PB with 0.5% Triton X-100. Next, sections were incubated overnight at room temperature with the appropriate combinations of primary antibodies at different dilutions (listed in Table 1) in PB with 0.5% Triton X-100. Sections were then washed in PB and incubated for 1 h with secondary antibodies. The secondary antibodies used were anti-rabbit-Alexa Fluor 488 and anti-mouse-Alexa Fluor 555 conjugates (1:100; Molecular Probes, Eugene, OR, USA). When indicated, the nuclear marker TO-PRO-3 iodide (Molecular Probes) was added at 1:1000. Finally, sections were washed in PB and mounted with Citifluor (Citifluor Ltd, London, UK) under a coverslip. Images were taken with a Leica TCS SP8 confocal laser-scanning microscope (Leica Microsystems, Wetzlar, Germany).

**Table 1:** Primary antibodies for immunofluorescence.

Molecular marker	Antibody	Supplier	Catalog No.	Dilution
Bassoon	Mouse monoclonal	Enzo	ADI-VAM-PS003	1:1000
Calbindin (CB)	Rabbit polyclonal	SWANT	CB-38a	1:500
Cone arrestin (CAR)	Rabbit polyclonal	MilliporeSigma	AB15282	1:200
GFAP	Mouse monoclonal	Sigma	#G3893	1:500
Iba-1	Rabbit polyclonal	Wako	#019-19741	1:1000
Peripherin-2 (PRPH2)	Rabbit polyclonal	ProteinTech	18109-1-AP	1:1000
PKCα	Mouse monoclonal	BD Transduction	610108	1:50
Rhodopsin (RHO)	Mouse monoclonal	MilliporeSigma	MAB5356	1:500
Synaptophysin (SYP)	Rabbit monoclonal	Abcam	ab52636-100	1:200

#### Electron microscopy

*Prph2*^*WT/WT*^ and *Prph2*^*KI/KI*^ mice at 1 month of age were perfused with a solution containing 4% paraformaldehyde and 2% glutaraldehyde in 0.1 M PB. Then, the eyes were enucleated and fixed for 2 h in the same solution. After rinsing in 0.1 M PB, the cornea, iris, lens, and vitreous body were removed, and the remaining posterior part of the eye was cut into four quadrants. They were postfixed in 1% osmium tetroxide (OsO_4_) in 0.1 M PB for 1 h and then gradually dehydrated in increasing concentrations of ethanol and acetone. The pieces were embedded overnight in EPON 812, and then blocks of EPON containing the pieces were made and polymerized at 60 °C overnight. Ultrathin sections were obtained from blocks and contrasted using lead citrate and uranyl acetate. Images were acquired with an FEI Tecnai Spirit BioTwin transmission electron microscope (Thermo Fisher Scientific) using Radius software v2.1 with an Xarosa digital camera (EMSIS GmbH, Münster, Germany).

#### Flow cytometry

Immune- and inflammatory-related cell populations in the retinas were analyzed by flow cytometry. After enucleating the eye, the retinas were dissected and disaggregated through a wide-bore pipette tip in 1 ml of pH 7.4 phosphate-buffered saline by carefully pipetting up and down. The retinal cell suspensions were then filtered to prevent clumps with a 30 mm strainer (BD Biosciences, San Diego, CA, USA). Samples were labeled with a cocktail of five antibodies acquired from e-Bioscience (San Diego, CA, USA): anti-CD11b-PE (Clone M/170), anti-CD45-FITC (Clone 30-F11) anti-CD11c-PerCpCy5.5 (Clone N418), anti-MHC class II (I-A/I-E)-PECy7 (Clone M5/114.15.2) and anti-CD169-eFluor 660 (Clone SER-4). After discarding doublets and debris events, the CD11b^+^ population (which stains macrophages and microglia cell populations) or the CD45^+^ cells (a marker of microglia and leukocyte populations) were selected [16–18]. The CD11b^+^ cell subpopulations were analyzed for their immunoreactivity against MHC class II (activated cells) [17, 19, 20], the dendritic cell marker CD11c (a marker of dendritic and microglia cell populations) [21–23] and sialoadhesin (CD169, which stains activated macrophages and microglial cell populations) [24–26]. Each mouse retina was analyzed individually, and 3–12 retinas from different animals were assessed for each experimental group. Data were acquired on an LSR Fortessa cytometer (BD Biosciences) and analyzed using FCS 6 Flow Cytometry Software (De Novo, Los Angeles, CA, USA).

#### Statistics

No statistical methods were used to predetermine the animal sample size. Data were statistically analyzed using GraphPad Prism Analysis Software (GraphPad Software, Inc., San Diego, CA, USA). Prior to the analysis of variance (ANOVA), data were tested for normality with a Kruskal-Wallis test, and homoscedasticity was assumed. ANOVA and Bonferroni’s post hoc test were applied to determine the significant differences between experimental groups. Differences were considered statistically significant at *P* < 0.05. Data in all graphs are represented as the mean value ± SEM.

## Results

### Generation of the C57BL/6J-*Prph2*^em1Sal^ animal model

**Fig. 1:**
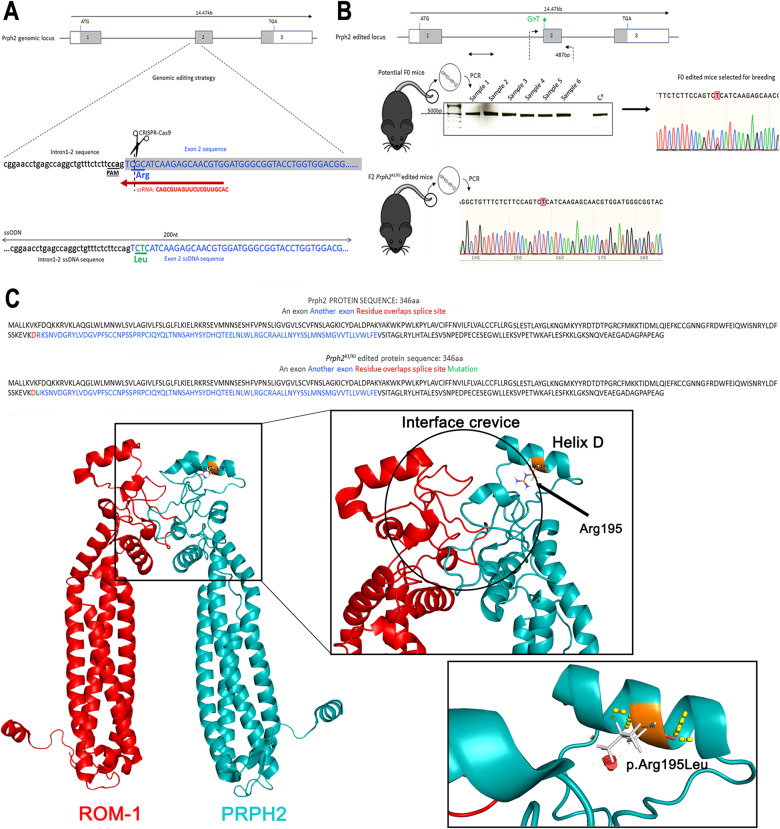
Generation of the central areolar choroidal dystrophy mouse model, C57BL/6J-*Prph2*^em1Sal^. **A** The genomic editing strategy consisted of the induction of a double-strand break at the c.584 position of the *Prph2* genomic *locus* using CRISPR/Cas9. An ssODN containing the mutation of interest was also injected as a template for DNA repair by a homologous recombination mechanism. **B** Potential founder mice (F0) were selected for breeding. PCR and Sanger sequencing were used to check that mice carried the c.584 G > T. **C** Modeling of wild-type and mutated proteins was performed with the PyMOL Molecular Graphics System (Version 2.0 Schrödinger, LLC New York, NY, USA) using the PDB file 7ZW1 [40] Steric interference is represented: large red disks show van der Waals overlap; short green lines represent atoms that overlap slightly or that are almost in contact.

The c.584 G > T knock-in mouse model was generated by homologous recombination using the CRISPR/Cas9 system in mouse zygotes (Fig. 1A). Pure *Prph2*^*WT/KI*^ animals were crossed to obtain *Prph2*^*KI/KI*^ mice. At this point, all animals were genotyped and then cross-bred to obtain the animals needed for the study. Genotyping showed the insertion of the point mutation in c.584 (Fig. 1B). This mutation resulted in the change of a single amino acid. Arginine at position 195, with a positively charged side chain, was exchanged by hydrophobic leucine. This mutation was localized in helix D, which is located in the peripheral area of the interface crevice, the domain involved in PRPH2 oligomerization (Fig. 1C). Retinal degeneration of both *Prph2*^*WT/KI*^ and *Prph2*^*KI/KI*^ mice was characterized at 1, 3, 6, 9, 12, and 20 months of age.

### Visual function decreases with age in peripherin mutant mice

**Fig. 2:**
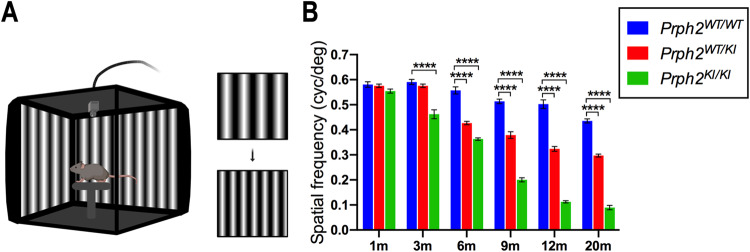
Decreased visual acuity in *Prph2* mutant mice. **A** Scheme of the optomotor system. The mouse is placed on an elevated platform, and visual stimuli are presented. Vertical gratings with different widths rotate clockwise or counterclockwise aleatory with a gradual decrease in spatial frequency. Smooth head and tail movements are evaluated by a trained observer. The visual acuity threshold corresponds to the highest spatial frequency to which the animal responded. **B** Evolution of visual acuity in heterozygous (*Prph2*^*WT/KI*^) and homozygous (*Prph2*^*KI/KI*^) *Prph2* mutant mice compared to control mice (*Prph2*^*WT/WT*^) from 1 to 20 months of age. Note that visual acuity decay in *Prph2*^*KI/KI*^ mice is more pronounced than that in *Prph2*^*WT/KI*^ animals. *P ≤ 0.05, **P ≤ 0.01, ***P ≤ 0.001, ****P ≤ 0.0001.

To assess the effect of the peripherin gene mutation on visual function, the VA of *Prph2*^*WT/WT*^, *Prph2*^*WT/KI*^, and *Prph2*^*KI/KI*^ mice was analyzed by optomotor test (Fig. 2A). As shown in Fig. 2B, the VA of both *Prph2*^*KI/KI*^ and *Prph2*^*WT/KI*^ peripherin mutant mice progressively decreased with age compared to that of *Prph2*^*WT/WT*^ mice, with higher decay in *Prph2*^*KI/KI*^ mice than in *Prph2*^*WT/KI*^ mice. Specifically, *Prph2*^*KI/KI*^ mutant mice showed significantly lower VA values than control mice from 3 months of age (28% reduction, *P* < 0.0001), and VA scores were minimal in these animals from 12 months of age (78% reduction, *P* < 0.0001). Meanwhile, *Prph2*^*WT/KI*^ mutant mice showed VA values significantly lower than those of *Prph2*^*WT/WT*^ mice from 6 months of age (24% reduction, *P* < 0.0001), and VA scores were still relatively high at 20 months of age (32% reduction, *P* < 0.0001).

**Fig. 3:**
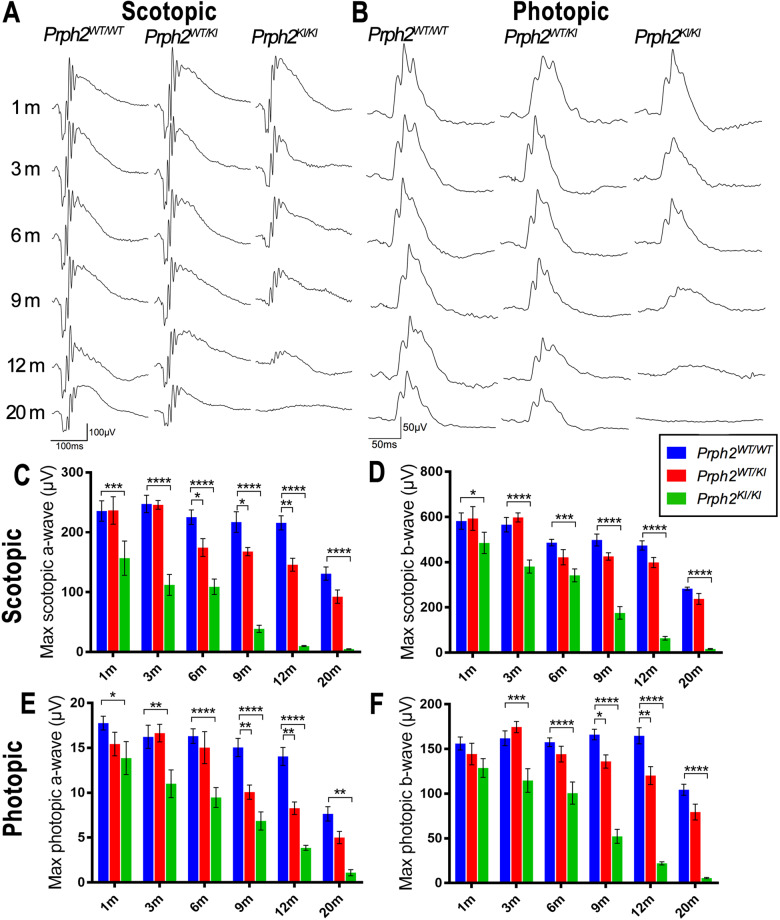
Age-dependent decrease in retinal responsiveness of C57BL/6J-*Prph2*^em1Sal^ mice. Representative maximum **A** scotopic (mixed rod-cone) and **B** photopic (cone-driven) electroretinogram responses in control (*Prph2*^*WT/WT*^), heterozygous (*Prph2*^*WT/KI*^), and homozygous (*Prph2*^*KI/KI*^) animals from 1 to 20 months of age. **C**–**F** Maximum amplitudes for both scotopic and photopic a- and b-waves in heterozygous (*Prph2*^*WT/KI*^) and homozygous (*Prph2*^*KI/KI*^) mice. *Prph2* mutant mice, compared to control (*Prph2*^*WT/WT*^) mice, from 1 to 20 months of age. Note that the decay in electroretinogram values was more pronounced in *Prph2*^*KI/KI*^ than in *Prph2*^*WT/KI*^ animals. *P ≤ 0.05, **P ≤ 0.01, ***P ≤ 0.001, ****P ≤ 0.0001.

Visual function in control and mutant mice was also assessed throughout ERG recording. Similar to the optomotor test results, ERG responses in both *Prph2*^*KI/KI*^ and *Prph2*^*WT/K*I^ mutant mice progressively diminished with age in comparison to those registered in *Prph2*^*WT/WT*^ mice, with the ERG response being more severely affected in *Prph2*^*KI/KI*^ mice than in *Prph2*^*WT/KI*^ mice (Fig. 3A, B). Specifically, the maximum scotopic a-wave values recorded in *Prph2*^*KI/KI*^ mutant mice significantly decreased from 1 month of age (34% reduction, *P* < 0.001) and practically disappeared from 9 months of age (83% reduction, *P* < 0.0001) (Fig. 3C). Meanwhile, the reduction in a-wave values in *Prph2*^*WT/KI*^ mice was significant from 6 months of age (33% reduction, *P* < 0.05), and a-wave scores remained noticeable at 20 months of age (Fig. 3C). Scotopic b-wave responses showed similar results, even though the drop in b-wave values was later and smoother than that observed in a-wave responses in both *Prph2*^*KI/KI*^ and *Prph2*^*WT/KI*^ mice (Fig. 3D). This indicates that the loss of function caused by *Prph2* mutation was earlier and more marked in the outer retina than in the inner retina. The later decline in the b-wave suggests that there is an adaptation to compensate for the decline in photoreceptor input. Photopic ERG responses also showed a progressive age-dependent diminution in a- and b-wave values of *Prph2* mutant mice compared to *Prph2*^*WT/WT*^ mice, with a more severe effect in *Prph2*^*KI/KI*^ than in *Prph2*^*WT/KI*^ mice and a slightly smaller effect on the photopic than on the scotopic response (Fig. 3E, F). This means that the point mutation in *Prph2* similarly affects the cone (photopic) response and the mixed (scotopic) response, with a slightly greater effect on the rod response than on the cone response.

### Structural and cellular alterations in the retinas of peripherin mutant mice

**Fig. 4:**
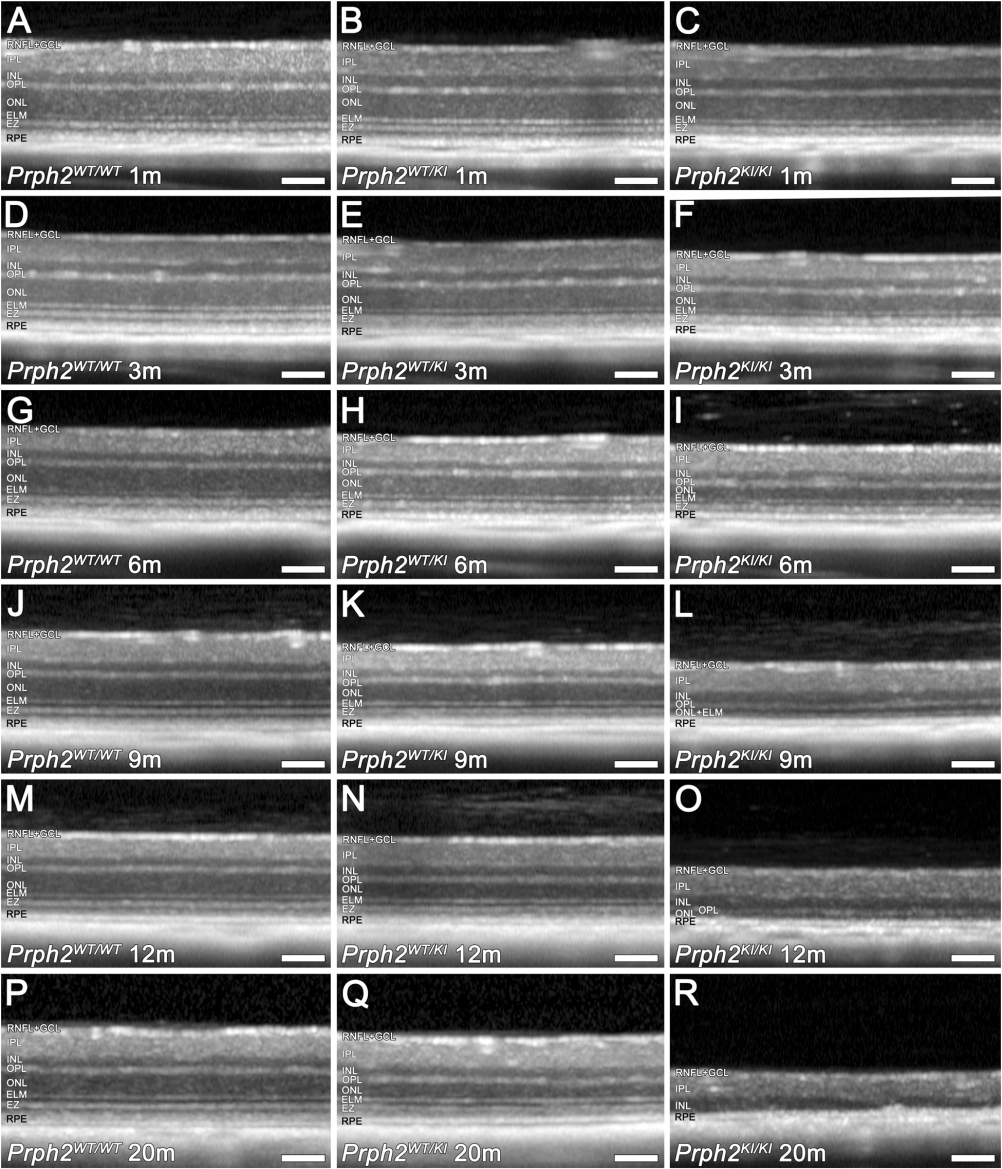
Structural alterations in the retinas of *Prph2* mutant mice evaluated in vivo by optical coherence tomography. Representative OCT images from Prph2 mutant mice from 1 to 20 months of age. Control (*Prph2*^*WT/WT*^) **A**, **D**, **G**, **J**, **M** and **P**; heterozygous (*Prph2*^*WT/KI*^) **B**, **E**, **H**, **K**, **N** and **Q**; and homozygous (*Prph2*^*KI/KI*^) **C**, **F**, **I**, **L**, **O** and **R**. RNFL retinal nerve fiber layer, GCL ganglion cell layer, IPL inner plexiform layer, INL inner nuclear layer, OPL outer plexiform layer, ONL outer nuclear layer, ELM external limiting membrane, EZ ellipsoid zone, RPE retinal pigment epithelium. Scale 100 µm.

**Fig. 5:**
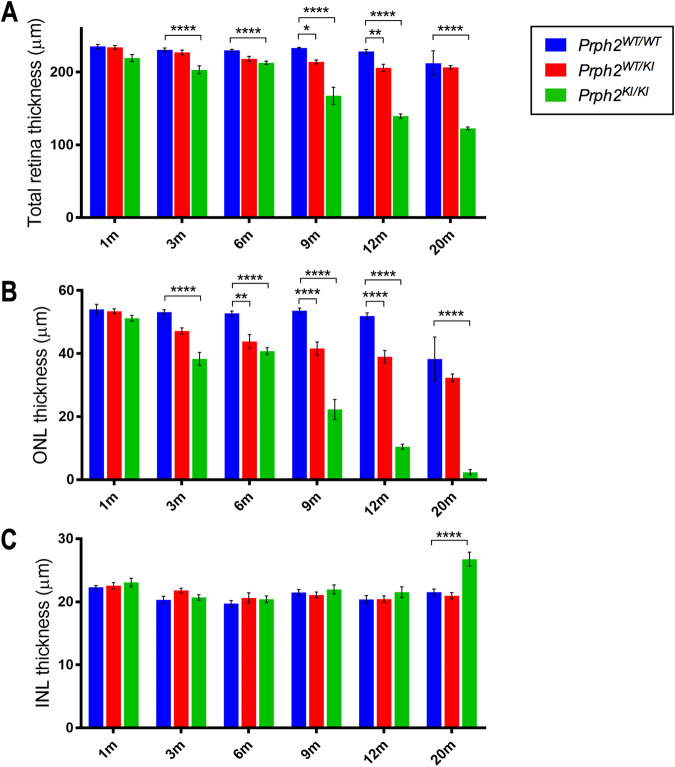
The outer nuclear layer thickness was significantly decreased in the C57BL/6J-*Prph2*^em1Sal^ model compared to wild-type animals. Retinal thickness was measured in images of control (*Prph2*^*WT/WT*^), heterozygous (*Prph2*^*WT/KI*^), and homozygous (*Prph2*^*KI/KI*^) mice from 1 to 20 months of age. **A** Total retinal thickness showed significant changes in the *Prph2*^*WT/KI*^ animals at 9 months of age and at 3 months for *Prph2*^*KI/KI*^ mice. **B** Outer nuclear layer (ONL) thickness was significantly decreased at 6 months in *Prph2*^WT/KI^ animals and at 3 months in *Prph2*^*KI/KI*^ animals. Indeed, this last model showed faster degeneration in ONL thickness than *Prph2*^*WT/KI*^ mice. **C** Inner nuclear layer (INL) thickness was not altered in the C57BL/6J-*Prph2*^em1Sal^ model.

OCT images were obtained from *Prph2*^*WT/WT*^, *Prph2*^*WT/KI*^, and *Prph2*^*KI/KI*^ peripherin mutant mice to study how the mutation affected the integrity of the retina. Figure 4 shows representative OCT images from each experimental group, and Fig. 5 shows the results of the measurement of total retinal, ONL, and INL thicknesses. In *Prph2*^*WT/WT*^ mice, there were no differences in the retinal structure over time. Although a decrease in the total retina and the ONL thickness was present in both Prph2 mutant mice over time, changes in the ONL thickness were more prominent. The INL remained stable among the experimental groups for up to 12 months (Fig. 4).

The total retinal thickness was significantly thinner at 9 months in *Prph2*^*WT/KI*^ mice (214 ± 3 µm, *P* < 0.05) and at 3 months in *Prph2*^*KI/KI*^ mice (203 ± 5 µm, *P* < 0.01) than in *Prph2*^*WT/WT*^ mice (3 months: 231 ± 3 µm, *P* < 0.0001; 9 months: 233 ± 1 µm, *P* < 0.0001) (Fig. 5A). While the alteration of the total retinal thickness in *Prph2*^*WT/KI*^ mice was smooth, the retina of *Prph2*^*KI/KI*^ animals showed a marked decrease at 9 and 12 months of age compared to that of *Prph2*^*WT/WT*^ mice. Differences in ONL thickness started to be significant from 6 months of age in *Prph2*^*WT/KI*^ mice and from 3 months in *Prph2*^*KI/KI*^ mice. Thus, these changes in total retinal thickness were mainly caused by the reduction in the ONL, since no changes were found in the INL among the experimental groups, except for an increase in this layer at 20 months in the *Prph2*^*KI/KI*^ mice (27 ± 1 µm, *P* < 0.0001) compared to the *Prph2*^*WT/WT*^ mice (21.5 ± 0.5 µm) (Fig. 5C). Unlike *Prph2*^*WT/WT*^ mice, the ONL of *Prph2*^*KI/KI*^ and *Prph2*^*WT/KI*^ mice continued to undergo degeneration over time. ONL thickness diminished progressively and smoothly in the *Prph2*^*WT/KI*^ retina with time (from 53.4 ± 0.8 µm to 32 ± 1 µm). Specifically, the ONL of *Prph2*^*KI/KI*^ mice showed marked changes at 3 (38 ± 2 µm, *P* < 0.0001), 9 (22 ± 3 µm, *P* < 0.0001), 12 (10.5 ± 0.8 µm, *P* < 0.0001) and 20 (2.4 ± 0.8 µm, *P* < 0.0001) months compared to *Prph2*^*WT/WT*^ animals.

**Fig. 6:**
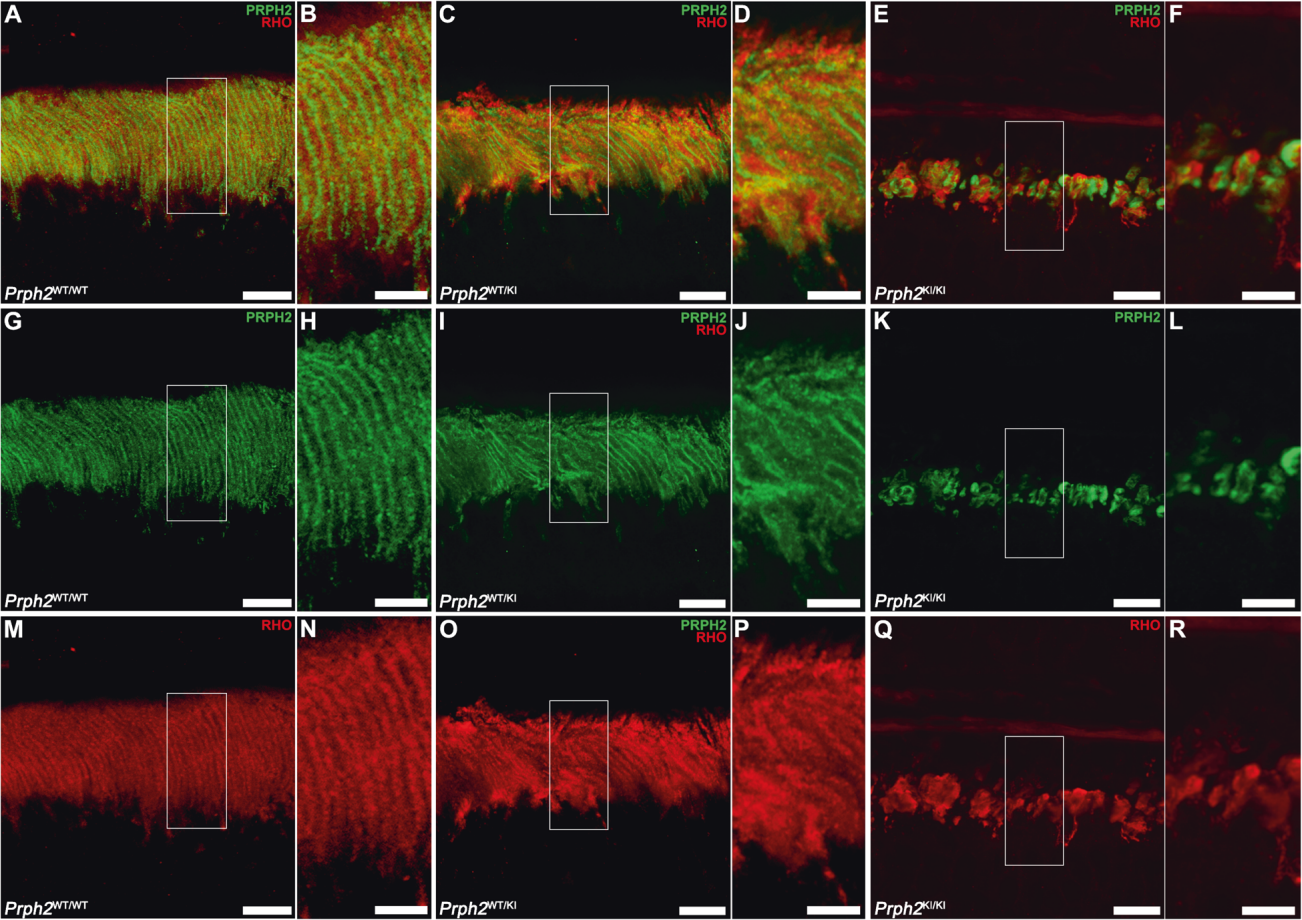
Outer segment structure was altered in the retina of the C57BL/6J-*Prph2*^em1Sal^ model. Peripherin was marked with anti-PRPH2 antibody (green) together with anti-rhodopsin antibody (RHO, red) to visualize the structure of outer segments in retinal cryosections of control (*Prph2*^*WT/WT*^), heterozygous (*Prph2*^*WT/KI*^), and homozygous (*Prph2*^*KI/KI*^) 9-month-old mice. Scale in (**A**, **C**, **E**, **G**, **I**, **K**, **M**, **O**, **Q**): 10 µm. Scale bars in (**B**, **D**, **F**, **H**, **J**, **L**, **N**, **P**, **R**): 5 µm.

**Fig. 7:**
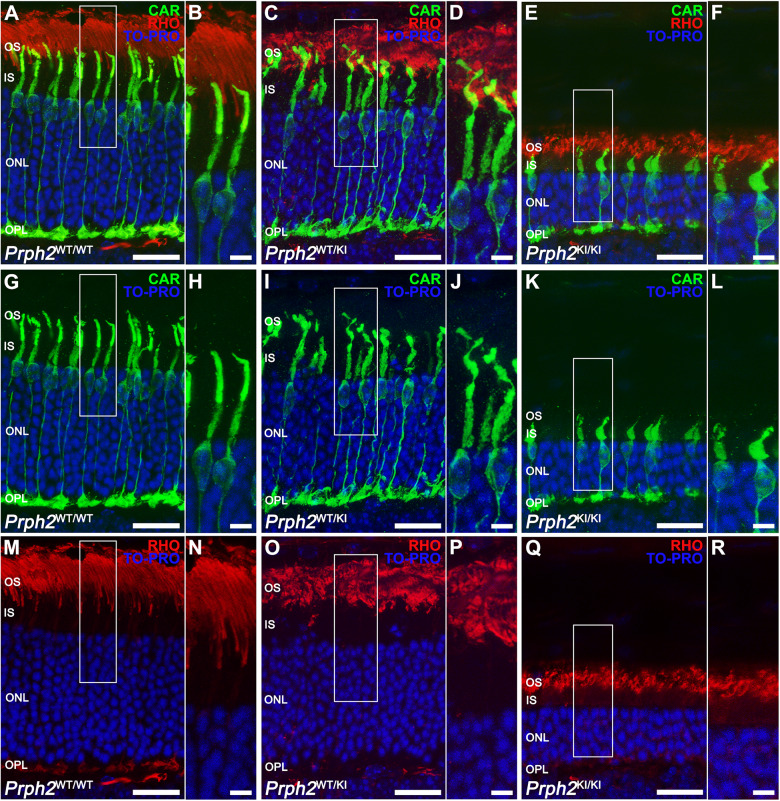
Morphological alterations in cones in the retinas of Prph2 mutant mice. Cones were immunolabeled with a cone arrestin (CAR, green) antibody to visualize their morphology. Anti-rhodopsin (RHO, red) antibody was added to mark the outer segments of cones. Nuclei were stained with TO-PRO-3 iodide (TO-PRO, blue). OS outer segment, IS inner segment, ONL outer nuclear layer, OPL outer plexiform layer. Scale in (**A**, **C**, **E**, **G**, **I**, **K**, **M**, **O**, **Q**): 20 µm. Scale bars in (**B**, **D**, **F**, **H**, **J**, **L**, **N**, **P**, **R**): 5 µm.

Since this mutation in *Prph2* affects visual function and the structural integrity of the retina, specific markers were used to study morphological changes in photoreceptor cells associated with the mutation. As PRPH2 is a structural protein required for the organization of the disc membranes, we expected that OSs were unstructured and, herein, that photoreceptor morphology was altered. To test this hypothesis, we performed immunolabeling in cryosections of the retinas of *Prph2*^*WT/WT*^, *Prph2*^*WT/KI*^, and *Prph2*^*KI/KI*^ mice at 9 months of age with anti-PRPH2 antibody to visualize OS morphology and possible mislocalization of the protein and anti-cone arrestin (CAR) antibody, which allowed us to visualize the entire morphology of cones, together with anti-rhodopsin (RHO) antibody, which immunolabels the OS of rods. Immunolabeling with anti-PRPH2 and anti-RHO antibodies showed well-structured OSs in the retinas of *Prph2*^*WT/WT*^ mice (Fig. 6A, B, G, H, M, N). PRPH2 immunostaining surrounded the immunolabeling of anti-RHO due to PRPH2 localization being restricted to the rim region of the discs (Fig. 6B), the specialized hairpin loop that circumscribed the two closely spaced membranes of each disc. In the retinas of *Prph2*^*WT/KI*^ animals, OSs looked swollen (Fig. 6C, D, I, J, O, P). Nevertheless, in the retinas of *Prph2*^*KI/KI*^ animals, there were membranous aggregations of OSs that were completely disorganized (Fig. 6E, F, K, L, Q, R). Additionally, RHO was mislocalized, which could indicate a loss of compartmentalization of the disc membranes (Fig. 6F, L, R). Immunolabeling with anti-PRPH2 also confirmed that mutant PRPH2 was not mislocalized toward the inner segment (IS) or the cell body in photoreceptor cells during the course of neurodegeneration and that the mutant protein remained confined in the OSs (Fig. 6D, J, P, F, L, R). Cone morphology was affected in the retinas of *Prph2*^*WT/KI*^ and *Prph2*^*KI/KI*^ mice, as shown by CAR immunostaining (Fig. 7). In the *Prph2*^*WT/KI*^ retina, the OSs of cones showed an unusual twisted appearance, losing the straight organization that is present in the OSs of the *Prph2*^*WT/WT*^ mouse retina (Fig. 7J). Pedicles also showed some morphological alterations in the *Prph2*^*WT/KI*^ retina, showing unusual telodendria. Cones in the retinas of *Prph2*^*KI/KI*^ animals were shortened, and their OSs were practically missing in contrast to those of *Prph2*^*WT/WT*^ animals (Fig. 7E, F, K, L). ISs of cones were swollen in the retina of the *Prph2*^*KI/KI*^ model. (Fig. 7E, F, K, L). Nuclear staining with TO-PRO-3 iodide confirmed the marked decline in ONL thickness in the retinas of *Prph2*^*KI/KI*^ mice observed with OCT, revealing a decreased number of photoreceptor rows (Fig. 7).

**Fig. 8:**
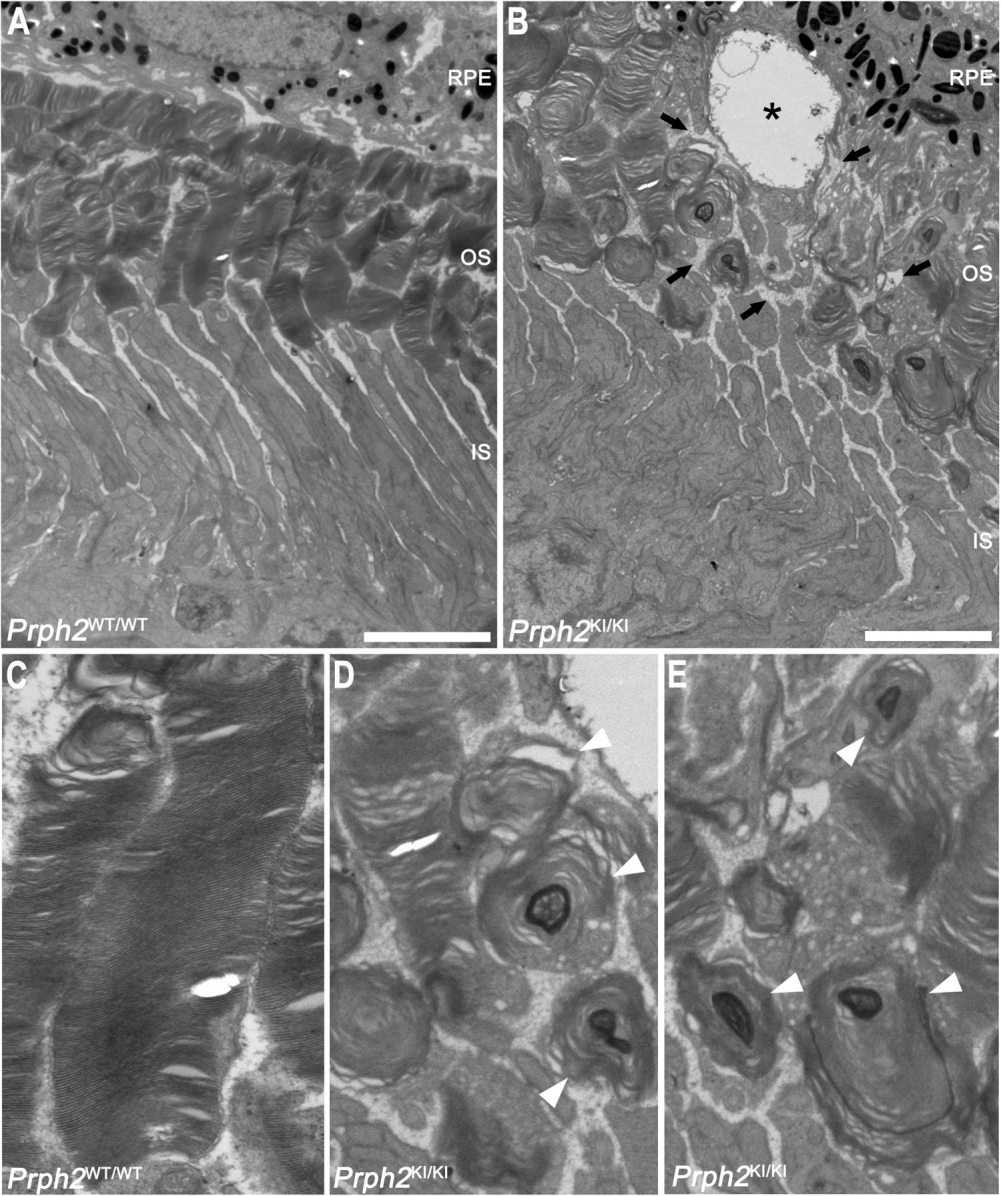
Transmission electron microscopy images of the retinas of wild-type and homozygous *Prph2* mutant mice. Images from **A** control (*Prph2*^*WT/WT*^) and **B** homozygous Prph2 mutant (*Prph2*^*KI/KI*^) 1-month-old mice and their respective magnifications (**C**–**E**). The black asterisk in (**B**) indicates an intracellular vacuole in an RPE cell. An increase in paracellular space was also observed and is indicated in the image with black arrows. **C** Detail of an outer segment of a photoreceptor cell from a *Prph2*^*WT/WT*^ animal. Disc membranes are perfectly organized, and the rim domain is clearly defined. **D**, **E** Magnification of concentric membranous circles (white arrowheads) found in the *Prph2*^*KI/KI*^ retina. RPE retinal pigment epithelium, OS outer segment, IS inner segment. Scale in (**A**, **B**): 5 µm. Scale in (**C–E**): 1 µm.

To deeply study the morphological changes in the OSs of photoreceptor cells in the C57BL/6J-*Prph2*^em1Sal^ model and to detect whether there were any structural alterations in the photoreceptor cells before detectable changes in the ERG or optomotor test, we performed transmission electron microscopy in the retinas of *Prph2*^*WT/WT*^ and *Prph2*^*KI/KI*^ mice at 1 month of age. At this age, OSs are still conserved in *Prph2*^*KI/KI*^. Because PRPH2 is necessary for the formation of photoreceptor discs and retinal immunostaining revealed a considerable impairment in OS structure, we decided to evaluate the ultrastructure of the OS discs. The *Prph2*^*KI/KI*^ mice displayed disorganized and shortened OSs and ISs compared with the *Prph2*^*WT/WT*^ mice (Fig. 8A, B). The normal parallel distribution of the OS disc enclosure in a membrane in *Prph2*^*WT/WT*^ mice (Fig. 8C) switched to major structural abnormalities of the OSs in *Prph2*^*KI/KI*^ mice (Fig. 8D, E). One of the most abnormal structures found in the OSs was a pattern of spirals or concentric circles that contained darker stained membranes (Fig. 8D, E, arrowheads). These structures were similar to those described in other peripherin mutations leading to the loss of visual function and photoreceptor degeneration found in this model [27, 28]. Additionally, we observed unusual intracellular vacuoles in the RPE (Fig. 8B, asterisk) and an increase in the paracellular space in the interphotoreceptor matrix (Fig. 8B, arrows). Both anomalies were also described by Tebbe and collaborators [29].

**Fig. 9:**
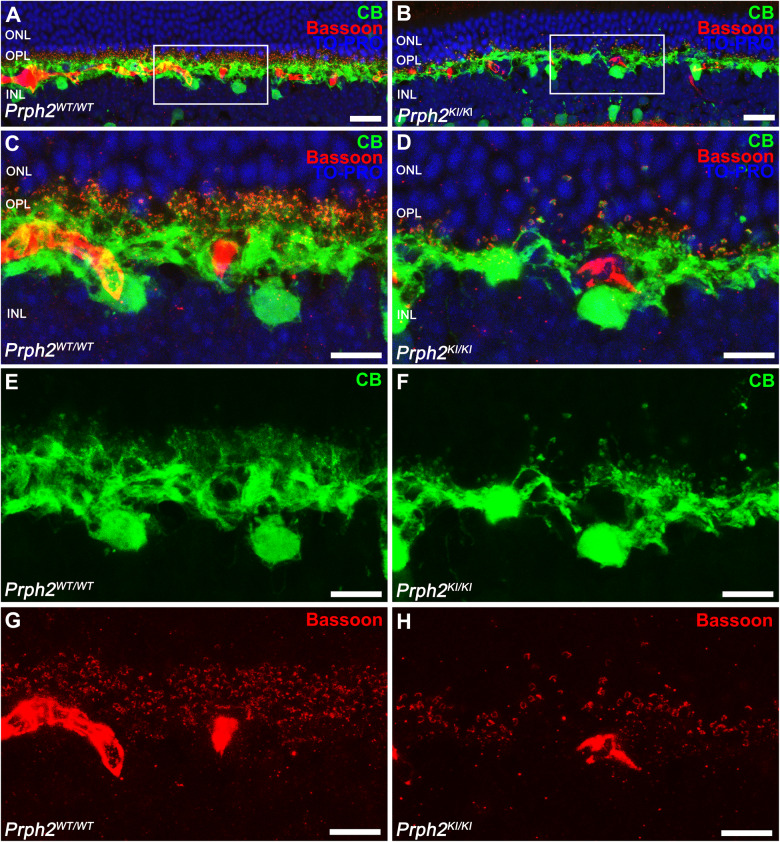
Loss of synaptic connectivity between photoreceptors and horizontal cells in homozygous *Prph2* mutant mice. **A**, **B** General view and **C**–**H** high magnification confocal images of retinal sections showing a dramatic decrease in synaptic contacts and dendritic arborization of representative horizontal cells after immunostaining with antibodies for horizontal cells (calbindin, green) and synaptic ribbons (bassoon, red) in the retinas of control (*Prph2*^*WT/WT*^) and homozygous (*Prph2*^*KI/KI*^) mice. Nuclei were stained with TO-PRO-3-iodide (TO-PRO, blue). OPL outer plexiform layer, ONL outer nuclear layer, INL inner nuclear layer. Scale bar in (**A**, **B**): 20 μm, scale bar in (**C**–**H**): 10 μm.

Considering the significant reduction in ONL thickness and photoreceptor nuclei in *Prph2* mutant mice, we proceeded to examine the synaptic connections in the retinas of both normal (*Prph2*^*WT/WT*^) and dystrophic homozygous (*Prph2*^*KI/KI*^) animals. Retinal sections were subjected to immunostaining using an anti-calbindin antibody, which specifically labels horizontal cells (Fig. 9A–F). Double labeling was performed with antibodies against bassoon, which highlights synaptic ribbons of photoreceptors (Fig. 9A–D, G, H). Images revealed a striking decrease in synaptic contacts between photoreceptors and horizontal cells. This decline in synaptic connectivity indicates that the mutation in *Prph2* not only affected the OSs of photoreceptors but also disrupted the interactions between photoreceptors and the second-order neural cells in the visual pathways. Consequently, this loss of synaptic connectivity in the OPL contributes to the overall deterioration of the visual system in *Prph2* mutant mice.

**Fig. 10:**
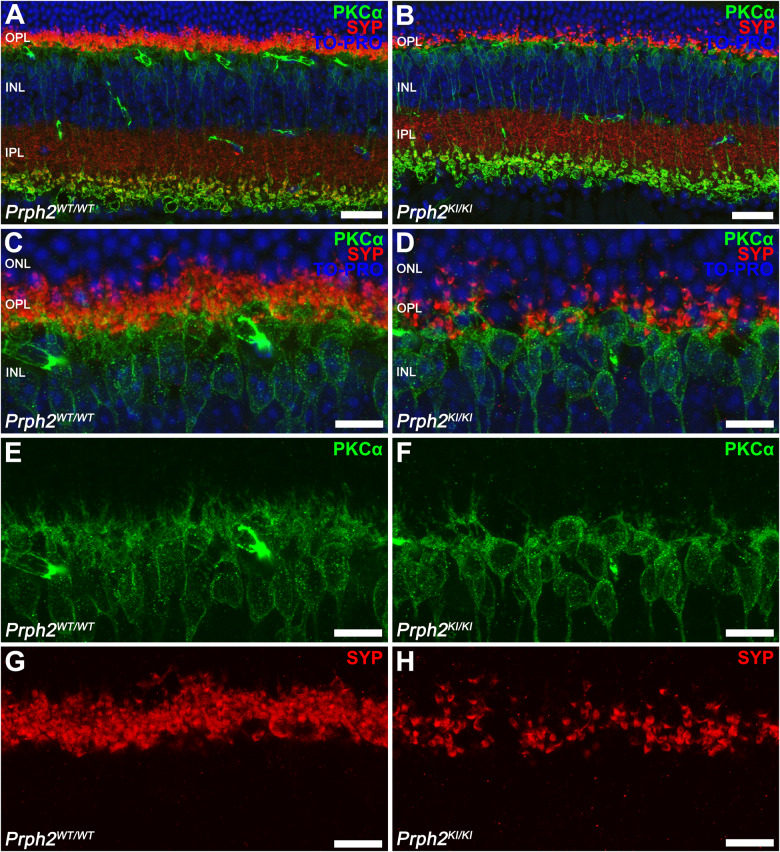
Analysis of ON-rod bipolar cells and their synaptic contacts with photoreceptors in C57BL/6J-*Prph2*^em1Sal^ mice. **A**, **B** Immunostaining was performed on retinal sections using antibodies against the α isoform of protein kinase C (PKCα, green) to label ON rod bipolar cells, while antibodies against synaptophysin were used to visualize synaptic vesicles in photoreceptors (SYP, red). **C**–**H** Higher magnifications of panels (**A**) and (**B**). Nuclei were stained with TO-PRO-3-iodide (TO-PRO, blue). Notably, the density of synaptophysin puncta associated with the dendritic tips of bipolar cells was higher in the retinas of control mice (*Prph2*^*WT/WT*^) than in those of homozygous (*Prph2*^*KI/KI*^) mice. ONL outer nuclear layer, OPL outer plexiform layer, INL inner nuclear layer, IPL inner plexiform layer. Scale bar in (**A**, **B**): 50 μm, scale bar in (**C**–**H**): 20 μm.

To investigate the synaptic contacts between photoreceptor cells and ON rod bipolar cells in the OPL, we conducted immunostaining of retinal sections using antibodies against synaptophysin, a specific marker of presynaptic vesicles. Additionally, double immunostaining with PKCα was performed to visualize the contacts between the axon terminals of photoreceptors and the dendrites of bipolar cells. In the retinas of *Prph2*^*KI/KI*^ mice, a noticeable reduction in synaptophysin-positive puncta (Fig. 10B, D, H) and a loss of bipolar cell dendrites labeled with PKCα (Fig. 10D, F) were observed compared to normal mouse retinas (Fig. 10A, C, E, G). Moreover, upon closer examination at high magnification of the double immunolabeling, fewer synaptophysin immunoreactive spots paired with bipolar cell dendritic tips were observed in the peripherin mice compared to the control (Fig. 10C, D).

**Fig. 11:**
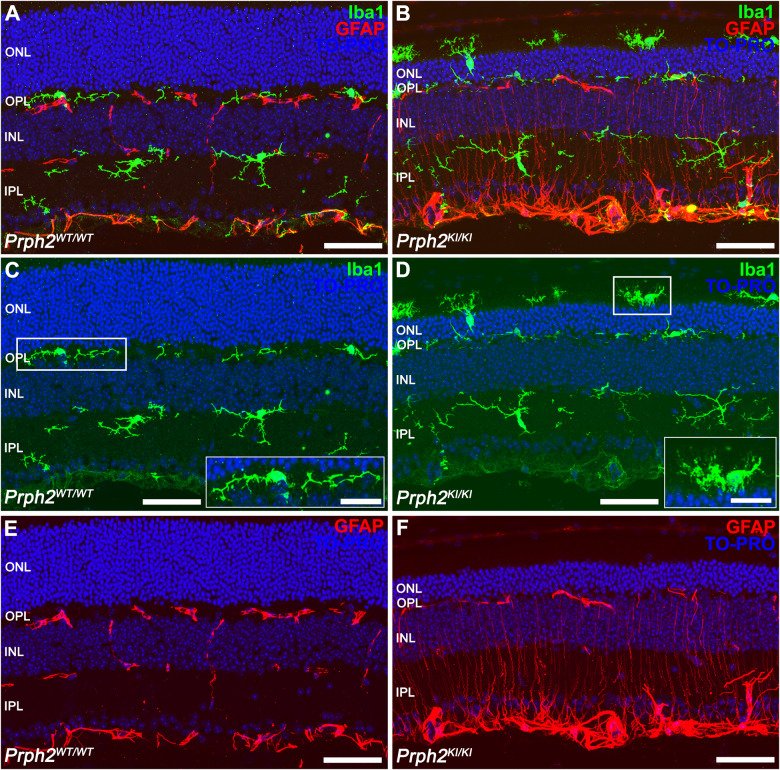
Microglial and Müller activation in *Prph2* mutant mice. **A**, **B** Representative vertical retinal sections immunostained with antibodies against Iba1 (green) to identify the morphology of microglial cells and GFAP to detect reactive Müller cells. **C**, **D** In a control healthy retina, resident microglial cells appear in the outer plexiform layer and the inner plexiform layer, with a ramified morphology. Nevertheless, when retinal degeneration exists, microglia show phagocytic activity with an ameboid morphology and can be found in the outer nuclear layer. **E**, **F** GFAP is expressed in astrocytes in the retinas of *Prph2*^*WT/WT*^ animals and in Müller cells in gliosis. Nuclei in the images were stained with TO-PRO-3-iodide (TO-PRO, blue). All images were captured in the central retina. ONL outer nuclear layer, OPL outer plexiform layer, INL inner nuclear layer, IPL inner plexiform layer. Scale bar: 50 µm. Scale bar in higher magnification images: 20 µm.

We also investigated the inflammatory process and the involvement of activated microglial cells in the C57BL/6J-*Prph2*^em1Sal^ model. For this purpose, we assessed the activity of Iba1^+^ cell populations in retinal sections (Fig. 11). Immunofluorescence images revealed an elevated presence of Iba1^+^ cells (shown in green) in the retinas of *Prph2*^*KI/KI*^ mice (Fig. 11B, D) compared to *Prph2*^*WT/WT*^ animals (Fig. 11A, C). Furthermore, these cells exhibited migration toward the photoreceptor layers, including the ONL and OSs, specifically in the retinas of *Prph2*^*KI/KI*^ mice. Conversely, in *Prph2*^*WT/WT*^ animals, Iba1^+^ cells were not detected above the OPL. *Prph2*^*KI/KI*^ mouse retinas exhibited an increased number of Iba1+ cells with an ameboid shape, indicating microglial activation in this particular model. To analyze the reactive gliosis of macroglial cells (astrocytes and Müller cells), we observed GFAP immunoreactivity. The retinas of *Prph2*^*KI/KI*^ mice demonstrated elevated GFAP immunoreactivity (Fig. 11B, F), which was higher than that in *Prph2*^*WT/WT*^ animals (Fig. 11A, E). Interestingly, the retinas of *Prph2*^*KI/KI*^ mice exhibited GFAP immunoreactivity not only at the inner margin but also throughout the entire length of Müller cells.

**Fig. 12:**
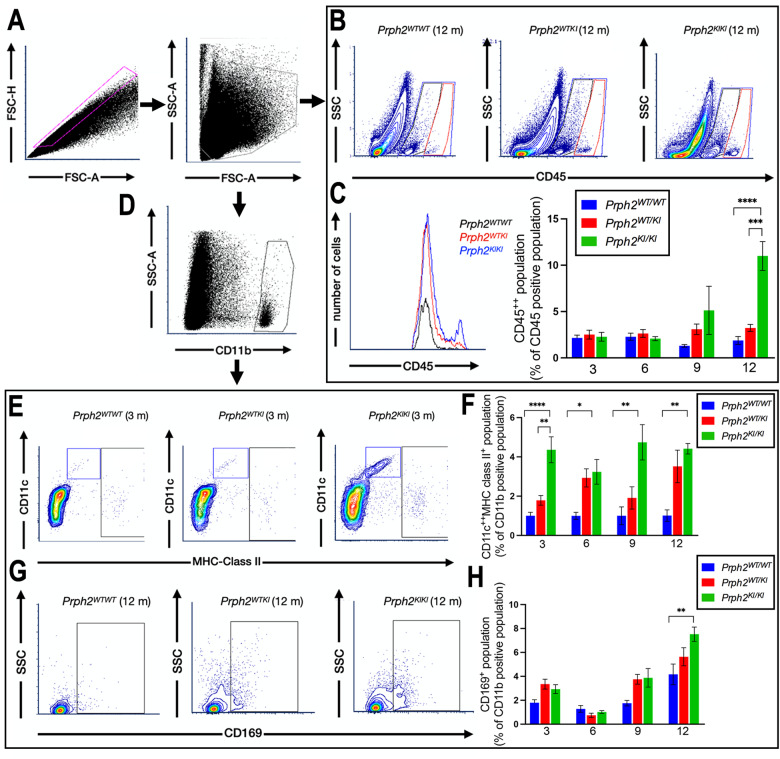
Phenotype of cell populations related to immune and inflammatory responses in *Prph2*^*WT/WT*^, *Prph2*^*WT/KI*^, and *Prph2*^*KI/KI*^ mouse retinas at different ages. Whole retinal cells from the three groups of mice were labeled with a cocktail of five antibodies: PE-conjugated anti-CD11b, FITC-conjugated anti-CD45, PerCpCy5.5-conjugated anti-CD11c, PECy7-conjugated anti-MHC class II (I-A/I-E), and eFluor 660-conjugated anti-CD169, and analyzed by flow cytometry. **A** Plots showing the gating strategy. Singlets were selected in a forward scatter (FCS)-H *vs* FCS-A dot plot, and cell debris was discarded in a side scatter (SSC)-A *vs* FCS-A dot plot. **B** CD45 medium- and CD45 high-immunoreactive cells were gated in contour plots representing SSC-A against CD45 immunofluorescence. Representative contour plots of the three different groups of mice at 12 months are shown. **C** Histogram showing CD45 immunofluorescence (merged values) of the three groups of mice at 12 months of age and bar graph presenting mean values ± SEM of the CD45 highly immunoreactive population (% of CD45^+^ population) in the three groups of mice at the different ages tested. **D** The CD11b^+^ population was gated in a dot plot (SSC vs CD45 immunofluorescence) and analyzed for reactivity against CD11c, MHC class II and CD169 antibodies. **E** Representative double dot plots presenting CD11c and MHC class II immunofluorescence of the CD11b^+^ population from the three groups of mice analyzed at 3 months of age. **F** Bar graph showing the mean values ± SEM of the CD11c^high^ MHC class II med population of the CD11b^+^ population in each group analyzed at different ages. **G** Representative dot plots presenting CD169 immunoreactivity of the CD11b^+^ population from the three groups of mice analyzed at 12 months of age. **H** Bar graph showing the mean values ± SEM of the CD169^+^ population (% of the CD11b^+^ population) in each group analyzed. All plots show data from a single representative experiment in each condition.

To analyze the inflammation- and immune-related cell populations in the different stages of the degenerative process, we examined the expression of several surface markers by flow cytometry in mouse retinas at different ages. After excluding doublets and cellular debris (Fig. 12A), CD45- or CD11b-immunopositive populations were selected (Fig. 12A, B). Among the CD45 immunoreactive cells (CD45^all^), two different populations were observed, one with medium reactivity (CD45^med^) and another highly immunopositive (CD45^high^) (Fig. 12B, C). The CD45^high^ population has previously been described as corresponding to an activated phenotype of microglia and macrophages, consistent with the invasion of peripheral cells [16–18, 30]. In *Prph2*^*KI/KI*^ mouse retinas, the percentage of the population with this phenotype was increased at advanced ages (9 and 12 months) and reached significance at 12 months of age (2.0 ± 1% in *Prph2*^*WT/W*^, 3.2 ± 0.7% and 11.0 ± 5% in *Prph2*^*WT/KI*^ and *Prph2*^*KI/KI*,^ respectively). In *Prph2*^*WT/KI*^ mouse retinas, the population was also increased with respect to the wild-type strain, although no significance was reached (Fig. 12D). CD11b^+^ cells were analyzed for their immunoreactivity against CD11c and MHC class II (Fig. 12E) or against CD169 (Fig. 12F) antigens. A significant increase in activated immune-related cells (CD11b^+^CD11c^high^MHC class II^+^) was observed in *Prph2*^*KI/KI*^ mice compared to control mice under all conditions tested (4 ± 2% to 1.0 ± 0.6% at 3 months, 3 ± 2% to 1.0 ± 0.6% at 6 months, 5 ± 2% to 1.00 ± 1% at 9 months, and 4.4 ± 0.6% to 1.0 ± 0.6% at 12 months) (Fig. 12E, G). In *Prph2*^*WT/KI*^ mouse retinas, the CD11b^+^CD11c^+^MHC class II^+^ population was increased, although no statistical significance was reached. The CD169 immunopositive population was significantly increased in *Prph2*^*KI/KI*^ mice at 12 months of age (7.53 ± 1.80%) with respect to *Prph2*^*WT/WT*^ and *Prph2*^*WT/KI*^ (4 ± 2% and 6 ± 1%, respectively) (Fig. 12F, H), a marker of pathogenic phagocytes [24, 25].

## Discussion

CACD is an inherited dystrophy that affects photoreceptors of the macula but also causes atrophy of choroidal vessels and RPE. Currently, there is no treatment for this disease, and the high heterogeneity of the phenotypes observed in patients, even in those who share the same mutation, makes it difficult not only to diagnose but also to establish the characteristics of the different stages of the disease [14]. The existence in the northern region of Spain of a family with 56 living patients with a p.Arg195Leu mutation in the *PRPH2* gene and with diagnosed CACD suggested that this mutation could serve as a good candidate for generating an animal model for this disease, which would serve us to effectively compare the evolution of the disease in the animal model and human patients. Hence, the generation of the new C57BL/6J-*Prph2*^em1Sal^ mouse allowed us to better understand the onset and progression of retinal degeneration in CACD. Moreover, having an animal model that reproduces the phenotype of an IRD associated with *rds/Prph2* will permit us to assay suitable neuroprotective therapies that could hopefully be extrapolated to humans in the future. Other animals with point mutations in *Prph2* were generated by other researchers [31–35], but to our knowledge, this is the first animal model carrying the mutation p.Arg196Leu, present in three large families, the abovementioned Spanish one, and in a Japanese and a German family [12, 36]. Moreover, this is the first study in which the onset and progression of PRPH2-associated retinal degeneration have been characterized over time by different techniques, and this is also the first time that synaptic remodeling of the OPL has been described in a model of IRD associated with *rds/Prph2*. We characterized the decay of retinal responsiveness and VA in this model in both heterozygosis and homozygosis. We also analyzed the alterations in the retinal structure of this model in vivo with OCT. Additionally, we studied the morphological alterations in photoreceptor cells and their OSs since PRPH2 is a key protein for the formation and organization of disc membranes. Additionally, we studied the loss of synaptic contacts with rod bipolar and horizontal cell dendrites using proper cell markers. Finally, we assessed the inflammatory state of the retina by studying Iba1^+^ cells and gliosis in Müller cells. We found a change in the phenotype of populations related to immune and inflammatory responses. In *Prph2*^*KI/KI*^ mouse retinas at 12 months of age, an increase in CD45^high^, CD11b, and CD169 immunopositive populations was observed, showing signs of microglial inflammation and/or recruitment of monocytes and macrophages [24, 25]. We also found an increase in the CD11b^+^CD11c^high^MHC class II^+^ population in *Prph2*^*KI/KI*^ mouse retinas at all ages tested. The expression of both integrin CD11c and MHC class II is increased in degenerating retinas [17, 19–23, 37]. The fact that this population is present even at young ages indicates that signals of inflammation are already present even when no signs of the disease are present, pointing to the potential benefits of early onset of the therapy and encouraging the study of early molecular changes that could be targeted therapeutically.

PRPH2 was first described as the product of the *retinal degeneration slow* (*rds*) gene. This gene is exclusively expressed in photoreceptor cells, and mice homozygous for *rds* show abnormal photoreceptor morphology and slow degeneration [38]. PRPH2 controls the precision of disc morphology, as it is key for the formation and organization of discs [7]. PRPH2 forms noncovalent homodimers and heterodimers by interacting with rod outer segment membrane protein 1 (ROM-1) in a head-to-head assembly. These dimers can be linked to form higher-order oligomers. Three dimers make up the scaffold of the rim region that circumscribes the disc membranes and, herein, orientates and stabilizes the OS discs [39–41]. How these complexes assemble into tertiary and quaternary conformations in the presence of mutated PRPH2 has a deep impact on disease onset and its phenotype [27]. According to other authors, incorrect oligomerization leads to the formation of discs that are too long and membranous accumulations that are denominated as “whorls” [27, 28, 42]. In this new model, we observed a marked disruption of photoreceptor OS morphology that affected both rods and cones, which is consistent with the decrease in both scotopic and photopic ERG responses in our mice. Transmission electron microscopy images revealed the “whorls” previously described by other authors, which consist of an accumulation of membranes in a concentric circular pattern [7, 27, 28, 39, 43].

The p.Arg195Leu point mutation of PRPH2 is located in helix D in the peripheral area of the interface crevice, which is implied in the assembly of the PRPPH2-PRPH2/ROM1 dimers. This mutation implies the change of a positively charged amino acid by a hydrophobic residue, which could provoke incorrect interactions of the side chains with the helix, such as alterations of the intrachain hydrogen bonding, that could cause changes in the dimer assembly at the interface crevice, provoking an imperfect arrangement of the rim scaffold. It has been reported that some point mutations of PRPH2 provoke the mislocalization of these proteins into the IS of photoreceptor cells, presumably due to retention in the endoplasmic reticulum [44, 45]. However, we did not observe PRPH2 accumulation in photoreceptor ISs or cell bodies. Herein, we hypothesized that the p.Arg195Leu mutation does not block PRPH2 transportation to the OSs of photoreceptor cells and its oligomerization. Therefore, disc membranes can be formed, although abnormally, as observed by immunolabeling of *Prph2*^*WT/KI*^ retinas. Therefore, we agree with previous researchers that different combinations of mutated and nonmutated PRPH2 oligomers can be formed in heterozygous models and patients [46]. Indeed, the protease cleavage assay performed by Böhm and collaborators showed that the p. Arg195Leu mutation does not disturb the overall folding of PRPH2 to a large degree [46]. Other models with point mutations in the region that participates in the dimerization of PRPH2 also showed the ability to initiate disc formation, but their morphology was aberrant [28]. In *Prph2*^*WT/KI*^ mice, there may be a mix of dimers formed by nonmutated and mutated proteins. Meanwhile, in *Prph2*^*KI/KI*^ animals, only aberrant dimers exist. Herein, this could explain the marked alteration of the morphological and visual function of the *Prph2*^*KI/KI*^ model compared to the *Prph2*^*WT/KI*^ model. Nevertheless, further experiments are needed to confirm this hypothesis. In any case, the morphological alteration in the disc membranes could trigger diverse processes of oxidative stress and inflammation, which can worsen the disease and lead to neurodegeneration with age [47–49].

In peripherin diseases, it is becoming increasingly evident that a single mutation can result in a range of different phenotypes, and conversely, it was observed that different mutations can lead to the manifestation of the same phenotype. This results in a strong heterogeneity of phenotypes, even within a family that shares the same mutation, with clinical manifestations ranging from retinitis pigmentosa to various forms of macular dystrophy [50, 51]. The presence of ROM1 variants [52], the existence of different PRPH2 haplotypes [53], and/or interactions with other genes/proteins [54] can modulate the patient phenotype. This intriguing pattern highlights the intricate relationship between specific genetic alterations and the resulting phenotypic expression. Consequently, a comprehensive investigation of these genotype-phenotype associations is imperative for gaining a deeper understanding of the underlying molecular mechanisms of the pathologies associated with PRPH2. Such research holds significant promise in unraveling the complex nature of these diseases and paving the way for more targeted and effective therapeutic interventions.

In a previous study by our group characterizing the phenotype of patients with the p. Arg195Leu mutation in *PRPH2*, we demonstrated that, even though different phenotypes were observed, all the patients showed a reduction of the ONL at the foveola and a disruption of the third outer retinal band, related to the outer segment tips [14]. These results agree with those obtained in our animal model, where we also observed a decrease in the ONL and alterations in the OSs in both rods and cones. Remarkably, the temporal progression of degeneration observed in human patients correlates with the time course of degeneration observed in mice, aligning the years of degeneration in a meaningful manner (6–9 mouse months are equivalent to 30–40 human years [55] when clear symptoms of the disease appear). Having an animal model that recapitulates the retinal degenerative process of the mutation can help to elucidate the cell death pathways involved in answering the question of how structural alterations of the OSs can activate cell death pathways, such as apoptosis, inflammation, or oxidative stress. This information is crucial for the development of targeted and effective therapies aimed at preventing or mitigating retinal degeneration.

In summary, since both functional and structural signs were observed in patients with the *PRPH2* mutation p.Arg195Leu were also found in C57BL/6J-*Prph2*^em1Sal^ mice, this animal model is the best approach to understanding the degenerative process of this retinal dystrophy and will permit us in the future to analyze the efficacy of different therapeutic approaches.

## Conclusion

In our investigation, the presence of the p.Arg195Leu point mutation in *Prph2* resulted in atrophy of OS disc membranes. Our study establishes a strong correlation between functional and morphological changes in the retinas of mutant mice. We observed a gradual decline in retinal thickness, with more pronounced and earlier alterations evident in homozygous mutants. Furthermore, novel findings from our study reveal impaired synaptic connectivity within the outer plexiform layer, along with the occurrence of Müller gliosis and microglial activation. As a result, the newly developed *Prph2*^*WT/KI*^ and *Prph2*^*KI/KI*^ murine models not only enhance our understanding of the mechanisms driving disease onset and progression but also offer reliable platforms for evaluating the effectiveness of innovative therapeutic strategies.

### Reporting summary

Further information on research design is available in the Nature Research Reporting Summary linked to this article.

### Supplementary information

Reporting summary

## Data Availability

The datasets generated during and/or analyzed during the current study are available from the corresponding authors upon reasonable request.
